# Multi-institutional noninvasive in vivo characterization of *IDH*, 1p/19q, and EGFRvIII in glioma using neuro-Cancer Imaging Phenomics Toolkit (neuro-CaPTk)

**DOI:** 10.1093/noajnl/vdaa128

**Published:** 2021-01-23

**Authors:** Saima Rathore, Suyash Mohan, Spyridon Bakas, Chiharu Sako, Chaitra Badve, Sarthak Pati, Ashish Singh, Dimitrios Bounias, Phuc Ngo, Hamed Akbari, Aimilia Gastounioti, Mark Bergman, Michel Bilello, Russell T Shinohara, Paul Yushkevich, Donald M O’Rourke, Andrew E Sloan, Despina Kontos, MacLean P Nasrallah, Jill S Barnholtz-Sloan, Christos Davatzikos

**Affiliations:** 1 Center for Biomedical Image Computing and Analytics, University of Pennsylvania, Philadelphia, Pennsylvania, USA; 2 Department of Radiology, Perelman School of Medicine, University of Pennsylvania, Philadelphia, Pennsylvania, USA; 3 Department of Pathology and Laboratory Medicine, Perelman School of Medicine, University of Pennsylvania, Philadelphia, Pennsylvania, USA; 4 Department of Radiology, University Hospitals Cleveland, Cleveland, Ohio, USA; 5 Penn Statistics in Imaging and Visualization Center (PennSIVE), Department of Biostatistics, Epidemiology, and Informatics, University of Pennsylvania, Philadelphia, Pennsylvania, USA; 6 Penn Image Computing and Science Lab (PICSL), University of Pennsylvania, Philadelphia, Pennsylvania, USA; 7 Glioblastoma Translational Center of Excellence, Abramson Cancer Center, Philadelphia, Pennsylvania, USA; 8 Case Comprehensive Cancer Center, Cleveland, Ohio, USA; 9 Department of Neurological Surgery, University Hospitals Seidman Cancer Center, Cleveland, Ohio, USA; 10 Department of Neurosurgery, Case Western Reserve University School of Medicine, Cleveland, Ohio, USA; 11 Department of Population and Quantitative Health Sciences, Case Western Reserve University School of Medicine, Cleveland, Ohio, USA

**Keywords:** gliomas, machine learning, molecular markers, open-source software, radio(geno)mics

## Abstract

**Background:**

Gliomas represent a biologically heterogeneous group of primary brain tumors with uncontrolled cellular proliferation and diffuse infiltration that renders them almost incurable, thereby leading to a grim prognosis. Recent comprehensive genomic profiling has greatly elucidated the molecular hallmarks of gliomas, including the mutations in *isocitrate dehydrogenase 1* and *2* (*IDH1* and *IDH2*), loss of chromosomes 1p and 19q (1p/19q), and epidermal growth factor receptor variant III (EGFRvIII). Detection of these molecular alterations is based on ex vivo analysis of surgically resected tissue specimen that sometimes is not adequate for testing and/or does not capture the spatial tumor heterogeneity of the neoplasm.

**Methods:**

We developed a method for *noninvasive* detection of radiogenomic markers of *IDH* both in lower-grade gliomas (WHO grade II and III tumors) and glioblastoma (WHO grade IV), 1p/19q in *IDH*-mutant lower-grade gliomas, and EGFRvIII in glioblastoma. Preoperative MRIs of 473 glioma patients from 3 of the studies participating in the ReSPOND consortium (collection I: Hospital of the University of Pennsylvania [HUP: *n* = 248], collection II: The Cancer Imaging Archive [TCIA; *n* = 192], and collection III: Ohio Brain Tumor Study [OBTS, *n* = 33]) were collected. Neuro-Cancer Imaging Phenomics Toolkit (neuro-CaPTk), a modular platform available for cancer imaging analytics and machine learning, was leveraged to extract histogram, shape, anatomical, and texture features from delineated tumor subregions and to integrate these features using support vector machine to generate models predictive of *IDH*, 1p/19q, and EGFRvIII. The models were validated using 3 configurations: (1) 70–30% training–testing splits or 10-fold cross-validation within individual collections, (2) 70–30% training–testing splits within merged collections, and (3) training on one collection and testing on another.

**Results:**

These models achieved a classification accuracy of 86.74% (HUP), 85.45% (TCIA), and 75.15% (TCIA) in identifying EGFRvIII, *IDH*, and 1p/19q, respectively, in configuration I. The model, when applied on combined data in configuration II, yielded a classification success rate of 82.50% in predicting *IDH* mutation (HUP + TCIA + OBTS). The model when trained on TCIA dataset yielded classification accuracy of 84.88% in predicting *IDH* in HUP dataset.

**Conclusions:**

Using machine learning algorithms, high accuracy was achieved in the prediction of *IDH*, 1p/19q, and EGFRvIII mutation. Neuro-CaPTk encompasses all the pipelines required to replicate these analyses in multi-institutional settings and could also be used for other radio(geno)mic analyses.

**Key Points**

- Radiogenomics model predicts *IDH* (85.45%), 1p/19q (75.15%), and EGFRvIII (86.74%) in gliomas.
- Pipelines provided by neuro-CaPTk can aid in therapeutic decision making on a patient basis.

**Importance of the Study**

Quantitative multivariate analysis of clinically acquired multi-parametric MRI reveals non-invasive in vivo imaging signatures of EGFRvIII, *IDH* and 1p/19q-codeletion in gliomas. The proposed approach differs from prior literature on the evaluation in a larger multi-institutional cohort, image analytic pipelines provided as part of neuro-CaPTk, and the application of a unified machine learning model for the assessment of different molecular markers. The current approach also differs on the extensiveness of images features, beyond what is customary in cancer imaging literature, used to quantify the structural and histological characteristics of the tumors, relating to tumor cell density, water content, and neo-vascularization. The discovered markers are derivatives of clinically available imaging sequences, therefore, can be rendered as readily translatable to the clinical practice, thereby eliminating the need of expensive molecular testing. An assessment of these markers at initial presentation or recurrence of the disease may facilitate personalized treatment planning, stratification into clinical trials, repeatable monitoring of molecular markers, and adoption of targeted therapeutic approaches.

Gliomas comprise a heterogeneous group of central nervous system tumors traditionally classified on a histologic basis. Over the past decade, with the advent of molecular profiling, there is a paradigm shift indicating that integrated histological–molecular classification is superior to a purely histological classification as highlighted in the 2016 World Health Organization classification of gliomas,^1^ where the definition of many of these gliomas now requires molecular characterization for their precise diagnosis. This fundamental change brings new challenges, one of which is to minimize disruption of current clinical practice, therapeutic trials, and epidemiological studies. These genomic characterizations have shown that mutations in the *isocitrate dehydrogenase 1* and *2* (*IDH1/2*) genes play a pivotal role in gliomagenesis, with a significant clinical and prognostic impact.^2^*IDH*-mutant gliomas are sub-divided into oligodendroglial and astrocytic types by the status of loss of chromosomes 1p and 19q, with the former presenting with distinctive morphology and better prognosis. Another important finding of the past decade demonstrates the association of epidermal growth factor receptor splice variant III (EGFRvIII) with triggering of various oncogenic processes eventually leading to aggressive tumor behavior,^3^ thereby making EGFRvIII a possible therapeutic target for high-grade gliomas.^4-6^ Hence, the evidence of the mutation’s presence can have an impact on treatment decisions, as well as on evaluating treatment response.

Currently, available techniques to determine molecular status vital for therapeutic decisions are immunohistochemistry and next-generation sequencing,^7^ which require tissue specimen analysis. These approaches are primarily limited by the sampling error, arising due to the spatial heterogeneity of the molecular landscape of gliomas.^8^ Furthermore, longitudinal assessment of these markers over the course of the treatment, and hence adaptation of the treatment accordingly, is not typically possible given the need for another invasive procedure. Some other limitations of the process include cases where collected tissue is inadequate for testing, tissue collection is not possible due to a deep-seated nature of tumors, or unavailability of expensive and/or specialized molecular assays in nonacademic settings.

The emerging field of radiogenomics exploits the data derived from complementary imaging modalities, enables noninvasive assessment of the various molecular features, and is increasingly used for oncologic diagnosis and treatment guidance. It involves the extraction of thousands of diverse and complementary quantitative imaging phenomic (QIP) features pertaining to volume, texture, morphology, kinetics, connectomics, intensity histograms, and spatial distributions of tumors. Integrating these QIP features via advanced computational methods allows for the identification of in vivo imaging signatures of molecular characteristics, enhances decision making, improves patient survival,^9–11^ and may transcend the limitations of one-size-fits-all treatment planning model, thereby leading to image-guided personalized treatment planning.

Despite increasing radiogenomics-based research^12^ and development of diagnostic and predictive biomarkers^12–14^ developed from these QIP signatures, they have yet not been adopted in routine clinical practice, in part due to their increasingly complex nature. Thus, there is a need for user-friendly software solutions, which can provide a bridge between novel radiogenomic research tools and their clinical applications, thereby enabling translation of cutting-edge research into practical and clinically useful diagnostic and predictive indices. Here we present one such imaging analytics suite, named neuro-Cancer Imaging Phenomics Toolkit (neuro-CaPTk), a general purpose tool spanning radiomics, radiogenomics, connectomics, and other research areas. Neuro-CaPTk is one component of CaPTk, which encompasses analytics suites for other oncologic conditions as well. Neuro-CaPTk is a modular platform, with components spanning image processing, segmentation, feature extraction, and machine learning (ML) that can be combined with the typical quantification, analysis, and reporting workflow of a neuroradiologist.

In this article, considering the relevant importance of *IDH* both in lower-grade gliomas and glioblastoma, 1p/19q in *IDH*-mutant lower-grade gliomas, and EGFRvIII in glioblastoma, we developed radiogenomic markers of *IDH*, 1p/19q, and EGFRvIII in the respective histologic categories. We present our results on a multi-institutional research study conducted by leveraging the QIP and ML routines provided by neuro-CaPTk to build imaging signatures of the aforementioned molecular markers. The contribution of our study arises from the evaluation in a larger multi-institutional cohort (*n* = 473), image analytic pipelines provided as part of neuro-CaPTk, and the assessment of different molecular markers using a unified ML model. An assessment of these markers at the initial presentation of the disease or at the time of recurrence may facilitate personalized treatment planning, enrollment of patients into clinical trials, and adoption of targeted therapeutic approaches.

## Materials and Methods

### Software, Hardware, and Pipeline Overview

**Figure 1.**
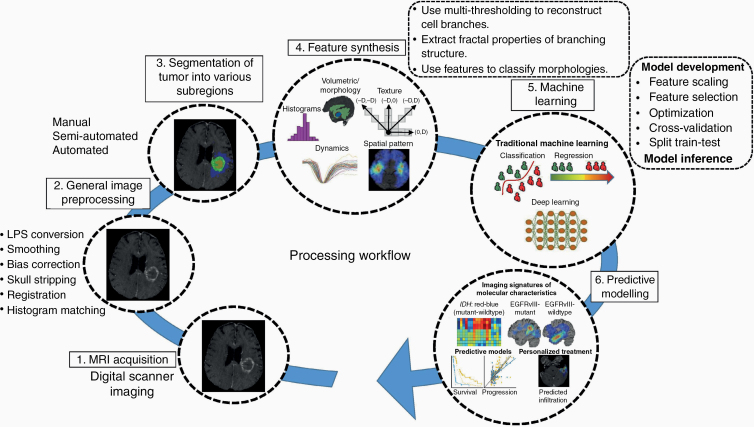
Detailed overview of the processing flow of neuro-CaPTk. The first tier (2, 3) provides basic image preprocessing routines. The second tier (4, 5) provides functionalities such as feature extraction and machine learning routines to integrate extensive QIP panel of features into algorithmically complex predictive and prognostic indices of interest. The third tier (6) provides specialized diagnostic and predictive models, leading to treatment planning and precision diagnosis, prediction of clinical outcome of interest, and noninvasive detection of molecular markers of tumors.

Neuro-CaPTk (www.cbica.upenn.edu/captk) has a 3-tier architecture (Figure 1). The first tier provides basic image preprocessing tasks such as image input–output (currently NIfTI and DICOM are supported), registration, and smoothing. The second level comprises various general purpose routines including feature extraction, feature selection, and ML. These routines are not only used within neuro-CaPTk for specialized tasks but are also available to the community as the basis for customized analysis pipelines. In particular, this level targets extraction of various features capturing different aspects of local, regional, and global imaging patterns, resulting in an extensive QIP panel which is compliant with the Image Biomarker Standardization Initiative (IBSI),^15^ selection of features to highlight smaller, meaningful feature sets from the larger ones, and finally, use of ML to build predictive and diagnostic models. The third level of neuro-CaPTk focuses on the integration of these features via ML algorithms provided within neuro-CaPTk toward specific goals, such as precision diagnostics,^14^ response assessment,^16^ and predictive models of survival^13^ (more details are given in Supplementary Section S1). Every specialized application within neuro-CaPTk is also available via the command line interface (CLI). These CLI applications can be called directly making them available as components within a larger pipeline or for efficient batch processing of large number of images. Neuro-CaPTk currently supports the visualization and image analysis of most of the important imaging sequences including structural magnetic resonance imaging (MRI), such as native (T1) and contrast-enhanced T1-weighted (T1-Gd), T2-weighted (T2), T2 fluid-attenuated inversion recovery (FLAIR), and advanced MRI such as dynamic susceptibility contrast MRI (DSC-MRI), dynamic contrast-enhanced MRI (DCE-MRI), and diffusion tensor imaging (DTI). In this study, neuro-CaPTk (commit hash 4f9688e) was used from the GitHub repository (https://github.com/CBICA/CaPTk).

Neuro-CaPTk is written in C++ using community-validated, open-source third-party libraries like the Insight ToolKit (www.itk.org), Visualization ToolKit (www.vtk.org), OpenCV (opencv.org), and Qt (www.qt.io; Supplementary Figure S1) and is fully cross-platform. The architecture and object-oriented development approach of neuro-CaPTk makes it easy to integrate new applications at the source level or as an external binary. Neuro-CaPTk extends to a cloud-based service to offer all the available algorithms through the public Image Processing Portal, which allows users to perform analyses using integrated algorithms, without any software installation, while using the high-performance computing resources of Center for Biomedical Image Computing and Analytics.

### Study Population

In this particular study, we utilized retrospective data with available preoperative MRI (T1, T2, T1-Gd, T2-FLAIR) from patients diagnosed with gliomas from 3 collections, all being part of the ReSPOND consortium^17^ (collection 1 [*n* = 248]: Hospital of the University of Pennsylvania [HUP]; collection 2 [*n* = 192]: The Cancer Imaging Archive [TCIA]; collection 3 [*n* = 33]: Ohio Brain Tumor Study^18,19^; Table 1). Data from collection 1 also had advanced MRI, including DSC-MRI and diffusion-weighted imaging (DWI), available. All MRIs of each patient were preprocessed using a series of image processing routines as detailed in “Image Preprocessing Operations Using Neuro-CaPTk” section. Molecular classification for the collections is described in Supplementary Section S2. All experiments were approved by the Institutional Review Board (IRB) of the HUP (approval no: 706564) and were carried out in accordance with the guidelines and regulations of the approved IRB and IRB approvals from each participating institution.

**Table 1.** Demographics of the Dataset Used in the Study (*n* = 473)

Characteristics	Collection 1 HUP (*n* = 248)		Collection 2 TCIA/TCGA (*n* = 192)		Collection 3 OBTS (*n* = 33)	
	Grade II–III	Grade IV	Grade II–III	Grade IV	Grade II–III	Grade IV
*Demographics*						
Age						
Mean ± SD	44.73 ± 11.75	60.14 ± 12.82	45.43 ± 13.69	58.10 ± 13.67	—	62.60 ± 11.38
Gender						
Male, *n*	5	141	44	55	—	21
Female, *n*	6	96	48	45	—	12
*Molecular markers*						
EGFRvIII mutation						
Available, *n*	—	213	—	—	—	—
EGFRvIII mutant, *n*	—	50	—	—	—	—
EGFRvIII wild type, *n*	—	163	—	—	—	—
*IDH* mutation						
Available, *n*	11 (0, 11)	74	92 (39, 53)	100	—	33
*IDH* mutant, *n*	10 (0, 10)	05	73 (35, 38)	05	—	07
*IDH* wild type, *n*	01 (0, 1)	69	19 (4, 15)	95	—	26
1p/19q codeletion						
Available, *n*	—	—	73^a^ (35, 38)	—	—	—
1p/19q codeleted, *n*	—	—	23 (11, 12)	—	—	—
1p/19q noncodeleted, *n*	—	—	50 (24, 26)	—	—	—

All the OBTS and HUP patients went through standard histopathological analysis (testing for *IDH* and 1p/19q) and they ended up being astrocytomous. Values within the parenthesis show number of grade II and grade III tumors, respectively. All the *IDH* mutations in HUP and OBTS datasets are *IDH1* mutation.

^a^All patients evaluated for 1p/19q status are *IDH* mutants.

### Image Preprocessing Operations Using Neuro-CaPTk

Preprocessing of MRI data involved various steps (Supplementary Figure S2), including (1) intensity noise reduction,^20^ (2) magnetic field inhomogeneity correction,^21^ (3) affine co-registration (6 degrees of freedom)^22^ between T1-Gd and the rest of the imaging sequences of each patient, and (4) skull stripping^23^ followed by manual revision when appropriate. Segmentation of tumors was carried out to identify tumorous subregions, that is, enhancing tumor (ET), nonenhancing portion of the tumor core (NC), and peritumoral edema/infiltrative tumor (ED),^24–26^ and neuro-CaPTk was used to manually annotate seed-points required for the initialization of segmentation process.^24,25^

Various derivative volumes such as peak height (PH), percent signal recovery (PSR), and an automatically extracted proxy to relative cerebral blood volume (ap-rCBV) (Supplementary Figure S4) were extracted from DSC-MRI scans using the routines provided by neuro-CaPTk. CaPTk also provides functionality to appropriately align DSC-MRI signals acquired across different institutions (Supplementary Figure S3). In addition, fractional anisotropy (FA), radial diffusivity (RAD), axial diffusivity (AX), and apparent diffusion coefficient (trace [TR]) were derived from DWI scans (Supplementary Figure S4).

### Feature Extraction

The preprocessed images were passed through the QIP feature extraction panel of neuro-CaPTk, which is designed based on an extensive panel of features compliant with the IBSI^15^ and is continuously evolving and serving as a general purpose toolkit for the community to quantify data characteristics. Relevant QIP features were computed for each patient from the 3 tumor subregions (ET, NC, and ED) and from all modalities, to capture phenotypic characteristics of various molecular markers. The extracted features include (1) multi-parametric imaging signals of different co-registered protocols/modalities; (2) volumetric measurements of different tumor subregions; (3) textural features (eg, from gray-level co-occurrence matrix,^27^ gray-level run-length matrix,^28^ gray-level size zone matrix,^28,29^ neighborhood gray-tone difference matrix,^30^ and local binary patterns^31^), quantifying characteristics of the local micro-architecture of tissue; (4) intensity distributions, reflecting various imaging signal distributions within the region of interest, the shapes of which convey functional and anatomical changes induced by the tumor; and (5) spatial distribution of tumor within an anatomical site of interest.^32^Supplementary Table S1 (.csv file) provides a detailed list of parameter values and can be directly used as input to neuro-CaPTk to extract the same set of features.

### Feature Synthesis and ML

A comprehensive set of QIP features extracted from various tumor subregions, including ET, NC, and ED, as detailed in “Feature Extraction” section, was integrated via ML modules provided by neuro-CaPTk to find the feature combination most predictive of molecular markers. The ML module in neuro-CaPTk is enabling users either to develop their own model on a given feature set and corresponding label set (in several well-known configurations such as k-fold, split-train-test) or to apply their existing model on a feature set to infer class information.

To confirm the robustness, accuracy, and generalizability of our method, while avoiding optimistically biased estimates of performance, we tested multiple configurations for the assessment of molecular markers in single- (configuration I) and multi-collection (configurations II and III) data.

#### Configuration I (single-collection data)

Here, the cohort of patients for *IDH* and 1p/19q molecular markers was randomly partitioned into discovery and replication (3:2 ratio) subsets. The ML model was trained on the discovery subset and validated on the replication subset. Split-train-test was not possible in EGFRvIII owing to the small number of EGFRvIII mutants compared to EGFRvIII wild types; therefore, we only applied 10-fold cross-validation for the detection of EGFRvIII in the HUP dataset. The *IDH*, 1p/19q, and EGFRvIII models were evaluated on lower-grade gliomas and glioblastoma, lower-grade gliomas, and glioblastoma, respectively.

#### Configuration II (multi-collection data: combined data from collections 1, 2, and 3)

This configuration was designed for an integrated cohort of 311 patients from the 3 collections having *IDH* status available. Discovery and replication subsets were selected the same way as in configuration I.

#### Configuration III (multi-collection data: collection 1 as discovery and collection 2 as replication)

This configuration was also specifically applied for the detection of *IDH*, wherein a model was trained on TCIA and then tested on HUP dataset.

In all the configurations, a Support Vector Machine (SVM) classifier with linear kernel was used to predict the molecular markers. The cost function of SVM was optimized on the discovery subset (9 fold in case of EGFRvIII) via 5-fold cross-validated grid search. To fit the SVM model, feature selection was performed using SVM forward feature selection on the discovery subset. The models trained on the discovery subset were then applied on the replication subsets in all the configurations, and the predicted scores were used for a receiver operating characteristic (ROC) analysis to measure the performance of the models.

## Results and Application

### Imaging Signatures of *IDH*, 1p/19q, and EGFRvIII

The evaluation of the models in configuration I yielded an accuracy of 86.74% (sensitivity = 84.91%, specificity = 87.50%, balanced accuracy [BAC] = 86.20%) in identifying EGFRvIII mutants in collection 1. Furthermore, the model demonstrated accuracies of 85.45% (sensitivity = 82.80%, specificity = 87.68%, BAC = 85.24%) and 75.15% (sensitivity = 81.49%, specificity = 73.96%, BAC = 77.73%) in identifying *IDH* and 1p/19q mutations, respectively, in collection 2 (Table 2).

**Table 2.** Performance of the Proposed Radiogenomics Pipeline for Detection of *IDH*, 1p/19q, and EGFRvIII Markers of Gliomas in Terms of Various Performance Measures

Configurations	Collection	Accuracy	Sensitivity	Specificity	Balanced accuracy (BAC)
Configuration I					
1p/19q codeletion	TCIA	75.15	81.49	73.96	77.73
*IDH* mutation	TCIA	85.45	82.80	87.68	85.24
EGFRvIII mutation	HUP	86.74	84.91	87.50	86.20
Configuration II					
*IDH* mutation	TCIA + HUP + OBTS	82.50	70.43	88.32	79.37
Configuration III					
*IDH* mutation	TCIA (discovery) + HUP (replication)	84.88	60.00	91.43	75.71

In configuration II, the proposed model when applied on the combined data of all the institutions for the prediction of *IDH* in split-train-test setting yielded a classification accuracy of 82.50% (sensitivity = 70.43%, specificity = 88.32%, BAC = 79.37%). In configuration III, the cross-validated classification success rate of 84.88% (sensitivity = 60.00%, specificity = 91.43%, BAC = 75.71%) was obtained in the detection of *IDH* mutation status when a model trained on all the patients of collection 2 was applied on collection 1.

**Figure 2.**
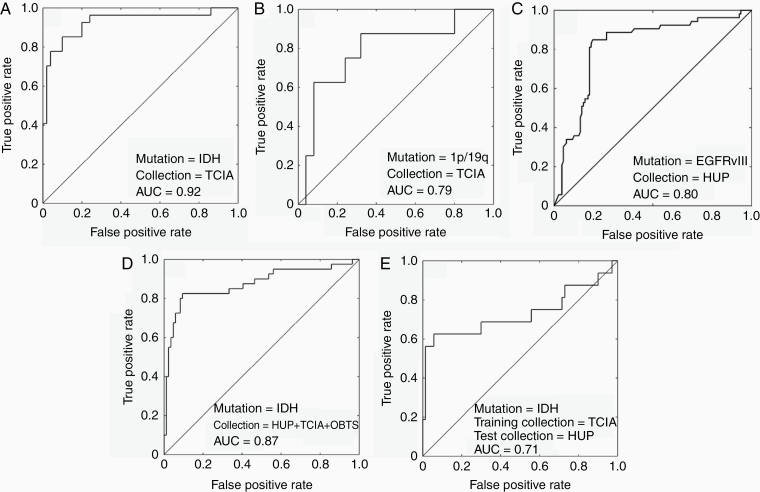
ROC curves of different experiments. Configuration I: A–C, configuration II: D, and configuration III: E.

ROC analysis was also used to illustrate the performance of the developed imaging signatures on an individual patient basis (Figure 2). The ROC curves were created by plotting the sensitivity against the false-positive rate (ie, 1-specificity) at various thresholds.

### Important Phenotypic Characteristics of Various Molecular Markers

**Figure 3.**
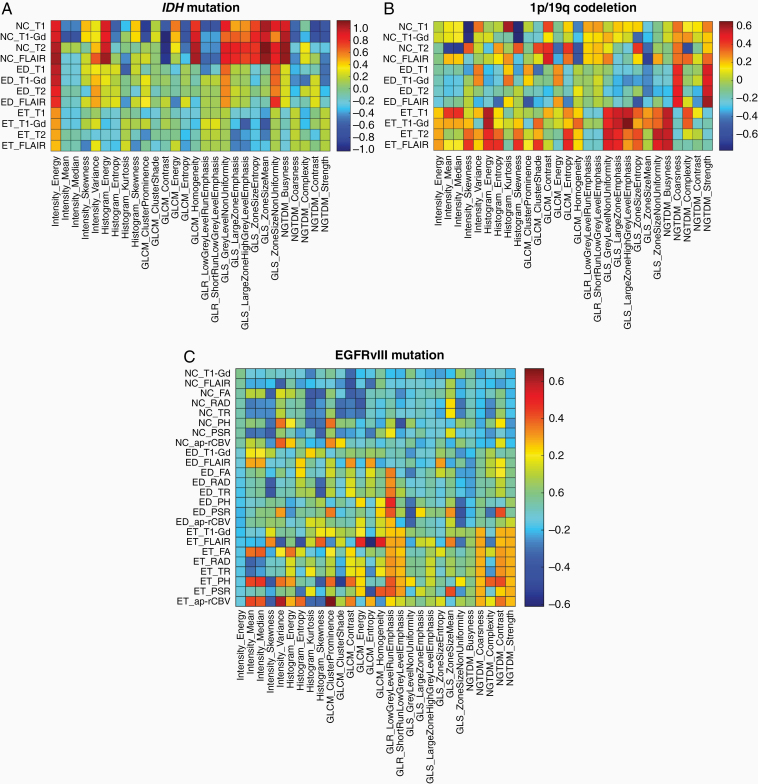
Effect sizes of the features used in the detection of (A) *IDH*, (B) 1p/19q, and (C) EGFRvIII. Red and blue colors indicate higher values of effect size, while red colors indicating features having higher values in category of interest (*IDH*-mutant, EGFRvIII-mutant, and 1p/19q-codeleted) and blue colors indicating features having higher values in the other category. Effect sizes in *IDH* category are shown for collection 2. GLCM = gray-level co-occurrence matrix,^27^ GLR = gray-level run-length matrix,^28^ GLS = gray-level size zone matrix,^28,29^ NGTDM = neighborhood gray-tone difference matrix.^30^

Considering the distinctive characteristics of different molecular markers and toward gaining an understanding about the biological processes associated with these distinctive characteristics, we sought to analyze each individual feature that we used to develop our ML predictive model. The effect size measure^33^ of important features is given in Figure 3.

EGFRvIII mutants presented imaging markers of neo-angiogenesis and cellular density, mainly represented by the elevated mean PH and mean rCBV value within ET, and decreased values of trace within NC. In the absence of advanced imaging for *IDH* analysis, the top-ranked features were T1-Gd and T1 intensity signals, both reduced in NC region, and texture features in NC region. Moreover, the features predictive of 1p/19q codeletion mainly represented features of spatial heterogeneity of all images in the ET region, all elevated in the 1p/19q-codeleted category.

### Spatial Distribution of the Tumors in the Brain

**Figure 4.**
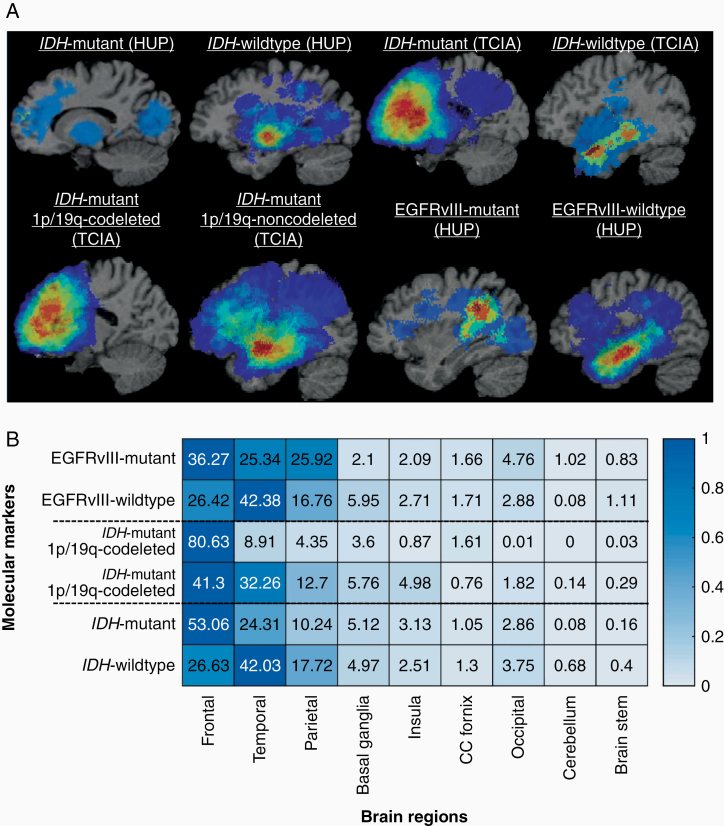
(A) Atlases representing distribution of tumors by *IDH*, EGFRvIII, and 1p/19q codeletion status. All images were displayed in the radiological convention orientation. (B) Percentage distribution of tumors in 9 brain regions by *IDH*, EGFRvIII, and 1p/19q codeletion status. Distributions were combined for *IDH* cases of HUP and TCIA.

Next, we created probabilistic atlases of tumor spatial distribution on a large cohort of glioma patients as a function of molecular markers, after taking into consideration mass effects properties from biophysical tumor growth models^34^ and deformable registration of tumor brain scans to a standardized anatomical atlas.^24,25^ We investigated whether the tumors pertaining to a particular molecular marker are distributed across all the regions or whether the underlying biological characteristics of these regions give rise to different mutational status. The *IDH*-mutant tumors had a clear predilection for the frontal lobe, especially on the left hemisphere. The *IDH*-mutant 1p/19q-codeleted tumors when compared with *IDH*-mutant 1p/19q-noncodeleted tumors were more frequently appearing in the frontal lobe. EGFRvIII-mutant tumors seemed to have a focused preference for frontal and parietal regions, and EGFRvIII-wild type tumors more frequently appearing in the temporal lobe. Occipital, brain stem, CC fornix, and temporal lobe were relatively less involved in the molecular markers under consideration (Figure 4).

## Discussion

This study investigated the use of in vivo MRI phenomic signatures leveraging ML for the prediction of molecular markers in glioma patients, aiming to offer advanced imaging biomarkers for clinical decision making and personalized treatment planning. Some important aspects that make our study unique compared to previously reported studies on prediction of molecular markers are the evaluation of radiogenomic markers in larger multi-institutional data, one unified model used to predict all the molecular markers, and an accompanying software tool (neuro-CaPTk) that encompasses all the pipelines required to replicate these analysis in multi-institutional settings. Most importantly, our results were derived via the utilization of neuro-CaPTk used to (1) extract rich clinically and biologically relevant features from MRI; (2) integrate imaging features via rigorous statistical and computational methodologies; and (3) train new ML models.

### Distinctive Characteristics of Radiomic Signatures

In this study, we designed multivariate radiomic signatures based on multi-parametric MRI for prediction of different molecular markers, including *IDH*, 1p/19q, as well as EGFRvIII. The main findings of the obtained radiomic signatures indicate that the set of features summarizing EGFRvIII mutants reflected lower TR and PSR, and higher ap-rCBV, PH, and FA compared to EGFRvIII-wild type tumors, consistent with prior studies.^35,36^ Radial and axial diffusivity measures showed similar trends as trace. In the previous study,^35^ however, a smaller cohort of 129 patients (compared to 213 patients in this study) was used, and the previous study relied on intensity, spatial location, and histogram binning-based features, without exploring the potential of advanced texture features. On the other hand, the current approach is based on 3 dimensional texture analysis for quantifying characteristics of the local micro-architecture of tissue, thereby capturing the entire tumor heterogeneity.

In the absence of advanced imaging modalities for the prediction of *IDH* and 1p/19q status, the algorithm mainly relied on features extracted from basic structural MRI modalities. Specifically, *IDH* mutants showed lower T1-Gd and T1 intensity signal in NC region, and higher homogeneity and lower entropy as reflected by the gray-level co-occurrence matrix-based texture features. In the case of *IDH*-mutant 1p/19q cases, the “entropy” and “non-uniformity” measures that both refer to the randomness in the region of interest were higher in the 1p/19q-codeleted group than in the 1p/19q-noncodeleted group, indicating that the tumors in the former group contained more regions with high gray levels, suggestive of higher radiologic heterogeneity.^37^ Other important features for accurate prediction of 1p/19q codeletion status were the location of the tumor, the features of the T2-weighted histogram, and other texture features. An important finding is that detection of *IDH*-mutants was more accurate in 1p/19q-codeleted tumors (5/5) compared to 1p/19q-noncodeleted tumors (18/22). Another interesting observation is that the spatial distribution of the tumors was one of the most distinctive feature of the molecular markers under investigation, therefore highlighting the importance of assessing spatial characteristics of tumors in a reference atlas template. Importantly, individual assessment of these features was not sufficient enough to identify the molecular markers (Figures 3 and 4); however, appropriate integration yielded sufficient accuracy for identifying the markers on an individual patient basis, thereby emphasizing the value of multivariate radiogenomic approaches.

Multiple recent studies have attempted to correlate T2–FLAIR mismatch with *IDH* and 1p/19q codeletion status in lower-grade gliomas.^38,39^ For example, MRIs of 125 lower-grade gliomas from the TCIA dataset were evaluated by 2 independent neuroradiologists to assess for the presence/absence of T2–FLAIR mismatch sign.^38^ All 15 cases declared positive by the readers for the T2–FLAIR mismatch sign were *IDH*-mutant 1p/19q-noncodeleted tumors. Extending upon this initial work, Foltyn et al.^39^ evaluated MRI scans of 408 glioma patients (113 low-grade and 295 glioblastomas) for the presence of T2–FLAIR mismatch sign by 2 independent reviewers. The T2–FLAIR mismatch sign was present in 12 low-grade gliomas, all of them being *IDH*-mutant 1p/19q-noncodeleted tumors, and was not found in any of the glioblastoma patients. These studies confirmed the high specificity of the T2–FLAIR mismatch sign for noninvasive detection of *IDH*-mutant 1p/19q-noncodeleted gliomas; however, sensitivity is low and applicability is limited to low-grade gliomas and glioblastomas. Moreover, the readers in these studies assessed all metrics in a qualitative and binary manner, and the inter-reader agreement was also low. The authors suggested that translation of this biomarker into a clinically applicable quantifiable prognostic measure would require extensive validation on larger datasets.^39^ In contrast, our study introduces simple and automated image-based assessment of molecular markers which appears to be highly accurate (*IDH:* specificity = 87.68%, sensitivity = 82.80%; 1p/19q-codeletion: specificity = 73.96%, sensitivity = 81.49%) of underlying molecular status.

Texture analysis is becoming a significant contributor to image quantification for more accurate, reliable, and objective medical diagnoses. It enables the quantification of image characteristics that are imperceptible to visual assessment, such as gray-level patterns, pixel interrelationships, and description of the variation in intensity within a specific area. When compared with various existing radiogenomic studies, our approach was based on 3-dimensional volumetric/texture analysis, thereby capturing the entire tumor heterogeneity, instead of either traditional image analysis without exploring the potential of advanced texture features,^35,40^ or texture analysis on a slice-by-slice basis, thereby not capturing the tumor heterogeneity in its entirety,^41,42^ or even on a limited number of slices, that is, 3 continuous MRI slices per subject used for the detection of 1p/19q status.^43^

In recent years, several deep learning (DL)-based approaches have gained popularity in the field of radiogenomics.^44,45^ Even though these methods have shown promise in the detection of *IDH* and 1p/19q status of gliomas,^44,45^ they tend to suffer with the problem of huge computational complexity. Also, these methods have a very large number of parameters and are therefore notorious for overfitting the data and suffering from poor reproducibility. Additional studies testing reproducibility of DL methods on multi-institutional data should be performed, before we can conclude that these methods are as promising as preliminary studies indicate. Furthermore, like conventional methods,^41,42^ some of the existing DL-based methods have also been applied to individual image slices, thereby, leading to underestimating the global texture within the tumor.^45,46^ Our method, on the other hand, captures complete heterogeneity of the tumor and provides almost similar performance on totally unseen test datasets with much lesser complexity and lesser chances of overfitting, therefore, we did settle with a standard pipeline of radiomic feature extraction, SVM forward feature selection, and classification.

### Clinical Relevance

Evaluation of molecular markers is currently typically done by analyzing tissue specimens, generally obtained from a single location within the tumor, via molecular based assays. This process has the following 2 limitations in determination of EGFRvIII status: (1) these molecular based assays destroy the tissue and invariably capture molecular markers using a small fraction of the tumor, thereby underestimating tumor heterogeneity and leading to sampling error; (2) repeated evaluation during treatment is not possible, due to invasiveness of the procedure, thereby limiting the measurement of temporal heterogeneity. In addition, the analysis of tissue specimens has some inherent limitations, applicable to all the molecular markers, such as limited tissue in case of inoperable and deep-seated tumors and in post-surgery follow-ups, or unavailability of expensive and/or specialized molecular assays in low-resource settings. Measures provided by imaging-based methods can therefore be critical in these cases. Our radiogenomic predictors could enable characterization of disease heterogeneity across the entire landscape of the tissue specimen and could be performed for a fraction of the price incurred in molecular testing. These predictors could also be helpful in cases where surgeons need to know the status of different molecular markers even before the resection, such as in case of neoadjuvant targeted therapies^47^ and the therapies that include intraoperative application of genotype-specific injections.^48,49^

While the current method focuses on *noninvasive* assessment of 3 molecular markers, *IDH*, 1p/19q, and EGFRvIII, the same method could also be used for assessment of other markers in general. Furthermore, the proposed signature can be evaluated to recurrent gliomas, with the goal of assessing molecular markers over the course of the treatment. This would help in *noninvasive* assessment of dynamic changes in the markers as response to targeted therapies (EGFRvIII in this case) and would in turn allow for tailoring the adopted therapeutic approaches.

The main contributions of our study arise from the evaluation of the pipeline in a larger multi-institutional cohort (*n* = 473), the assessment of different molecular markers using a unified ML model, and extraction of physiological (intensity and texture features) and anatomical properties of tumors beyond what is customary in cancer imaging literature. The imaging-based signatures proposed in this work are derivatives of MRI sequences that are acquired routinely according to current standard practice for gliomas, therefore, can be rendered as readily translatable to the clinical practice. Most importantly, the unique aspect of our study that makes it distinct among all the existing radiogenomic studies is the availability of the imaging-based pipelines used in this study via neuro-CaPTk that facilitates the prediction of the molecular markers shown in this study, as well as other markers. Neuro-CaPTk may be used in different facilities for the purposes of research, diagnosis, and education and may also be particularly useful for collecting “second opinions” on challenging cases.

### Limitations and Future Work

Our study has several limitations. Some of the ML models developed here have been built on small populations (such as 1p/19q), therefore, may not generalize well on new unseen populations of diverse background than those represented by the provided data. We expect the performance to improve as we increase the number of subjects and add more multi-institutional data in the training process. One more limitation of our study is that we used retrospective multi-institutional data; a prospective dataset comparing our methods to standard histopathological review would lend further validity and confidence to our ML models. Future work would include the creation and validation of ML models through the neuro-CaPTk application for various other molecular characterizations, including transcriptomic subtypes,^50^ as well as detection of other distinct molecular markers (eg, PTEN, TP53, and ATRX). Moreover, enthusiastically taking on the evolving field of integrated diagnostics, we aim to provide comprehensive diagnostic modules integrating radiology, pathology, and clinical markers in neuro-CaPTk.

## Conclusions

Our results imply that imaging signatures developed using radiomic models could predict molecular markers in gliomas. These predictions may contribute to the upfront assessment of molecular markers in neuro-oncological conditions for patients with inadequate tissue/inoperable tumors and earlier stratification of patients into clinical trials prior to acquisition of tissue-based molecular testing results. The extensibility of the radiogenomic modules incorporated in neuro-CaPTk, coupled with the flexibility afforded by programmatic construction of pipelines, facilitates the design of comprehensive analyses across a wide range of research studies and sites.

## Funding

This study was supported by the National Institutes of Health and Informatics Technology for Cancer Research grants R01-NS042645 and U24-CA189523.

## Authorship statement:

S.R.: Conceptualization, methodology, CaPTk programming, data interpretation, validation, analysis, data collection, writing, and editing; S.M.: data interpretation, validation, analysis, writing, and editing; S.B.: data interpretation, validation, analysis, writing, and editing; C.S.: methodology, data interpretation, validation, analysis, data collection and preprocessing, writing, and editing; C.B.: data interpretation, validation, analysis, data collection, writing, and editing; S.P.: CaPTk programming, data interpretation, validation, writing, and editing; A.S.: CaPTk programming, data interpretation, validation, writing, and editing; D.B.: CaPTk programming, data interpretation, validation, writing, and editing; P.N.: CaPTk programming, data interpretation, validation, writing, and editing; H.A.: data interpretation, validation, writing, and editing; A.G.: data interpretation, validation, writing, and editing; Ma.B.: data interpretation, validation, writing, and editing; Mi.B.: data interpretation, validation, analysis, data collection, writing, and editing; R.T.S.: data interpretation, validation, analysis, writing, and editing; P.Y.: data interpretation, validation, analysis, writing, and editing; D.M.O.: data interpretation, validation, analysis, data collection, writing, and editing; A.E.S.: data interpretation, validation, analysis, data collection, writing, and editing; D.K.: data interpretation, validation, analysis, writing, and editing; M.P.N.: data interpretation, neuropathology, validation, analysis, data collection, writing, and editing; J.S.B.-S.: data interpretation, validation, analysis, data collection, writing, and editing; C.D.: conceptualization, methodology, data interpretation, validation, analysis, writing and editing, supervision, and funding acquisition.

**Conflict of interest statement.** S.M. has received consulting income from Northwest Biotherapeutics, with research grants paid to the institution from Novocure, and Galileo CDS. R.T.S. has received consulting income from Genentech/Roche and compensation for scientific review from the American Medical Association, Research Square, and the Emerson Collective. The other authors made no disclosures.

## Supplementary Material

vdaa128_suppl_Supplementary_Material

vdaa128_suppl_Supplementary_Table-S1
