# Supplementary material for: Multi-institutional noninvasive in vivo characterization of IDH, 1p/19q, and EGFRvIII in glioma using neuro-Cancer Imaging Phenomics Toolkit (neuro-CaPTk)
Source: Neurooncol Adv. 2021 Jan 23;2(Suppl 4):iv22–34. doi: 10.1093/noajnl/vdaa128 (PMC7829474; doi:10.1093/noajnl/vdaa128)
Supplement: vdaa128_suppl_Supplementary_Material [file vdaa128_suppl_supplementary_material.docx]

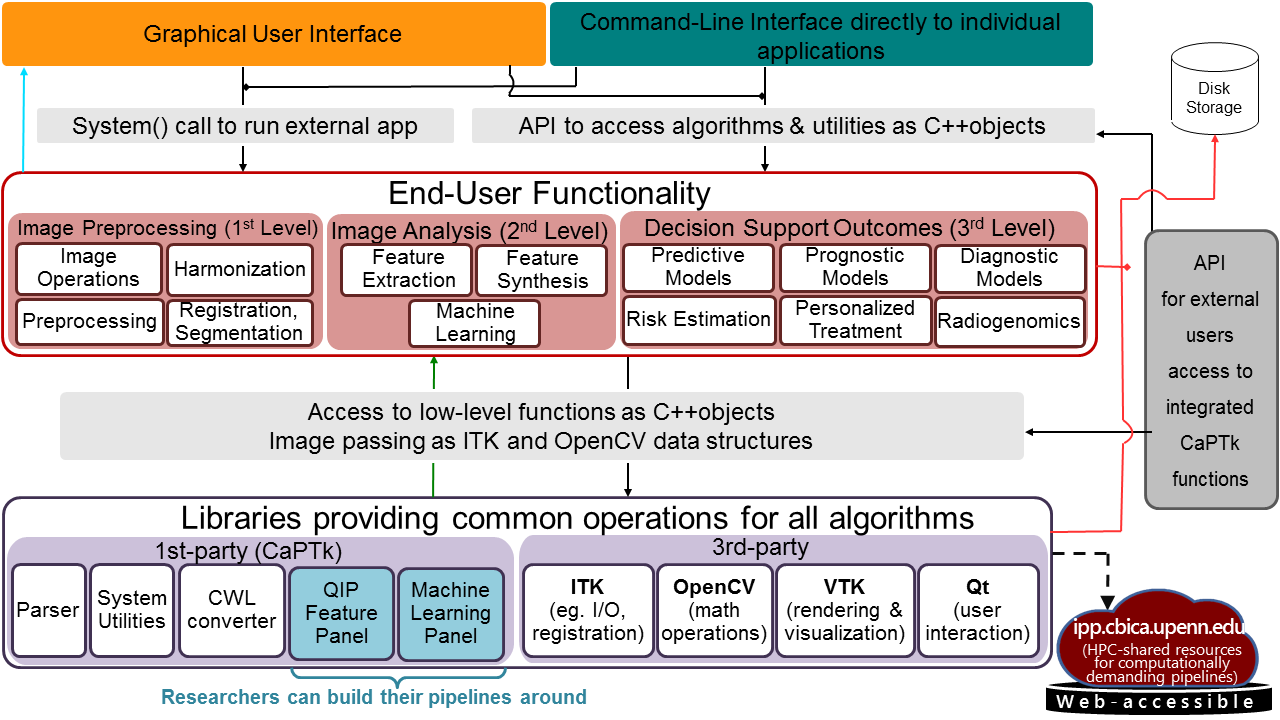


**Figure S1.** An overview of the neuro-CaPTk software architecture, including interfaces to external libraries, such as ITK, VTK and OpenCV, making it relatively easy for developers to integrate their algorithms, as well as connections to internally-developed algorithms and models.

**
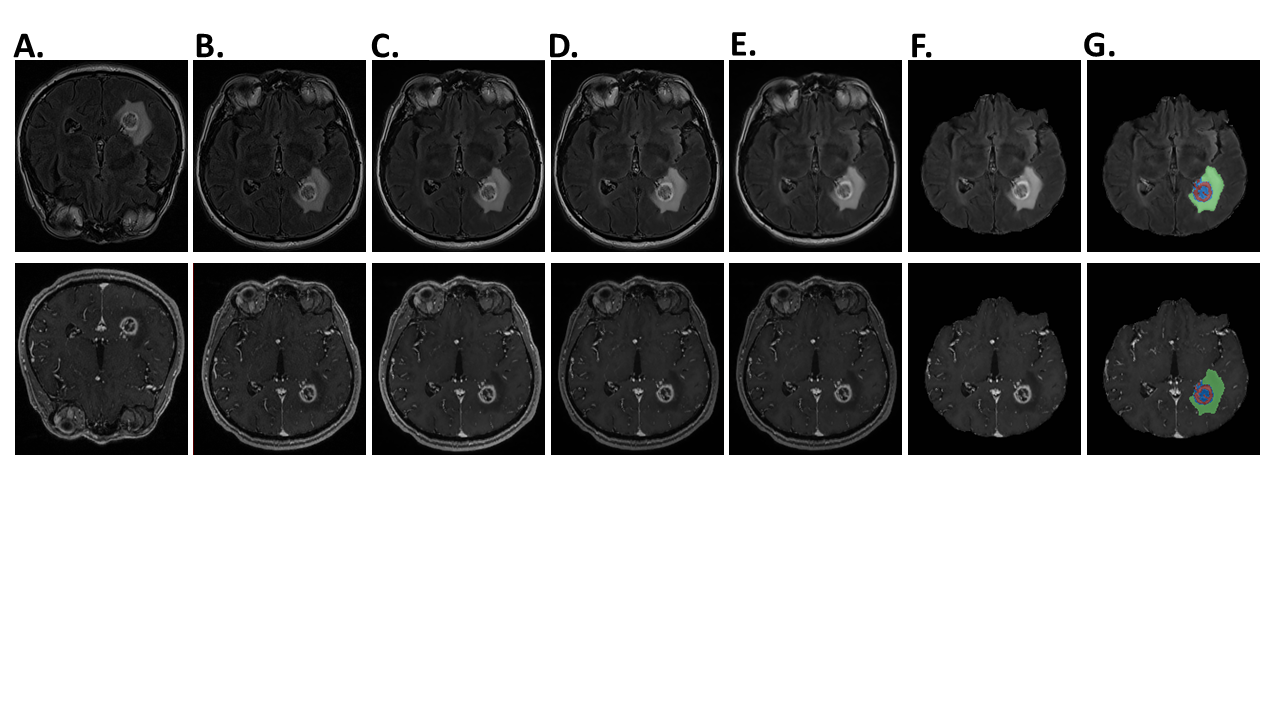
**

**Figure S2.** Regular preprocessing steps applied on brain tumor MRI scans, T2-FLAIR (top-row) and T1-Gd (bottom-row): (A) Raw DICOM, (B) Conversion to LPS format, (C) Susan denoising, (D) Bias correction, (E) Registration of FLAIR image with the corresponding T1-Gd image, (F) Skull Stripping, (G) Segmentation of brain tumor into various sub-regions, including enhancing tumor (red), non-enhancing tumor core (blue), and peri-tumoral edema region (green).


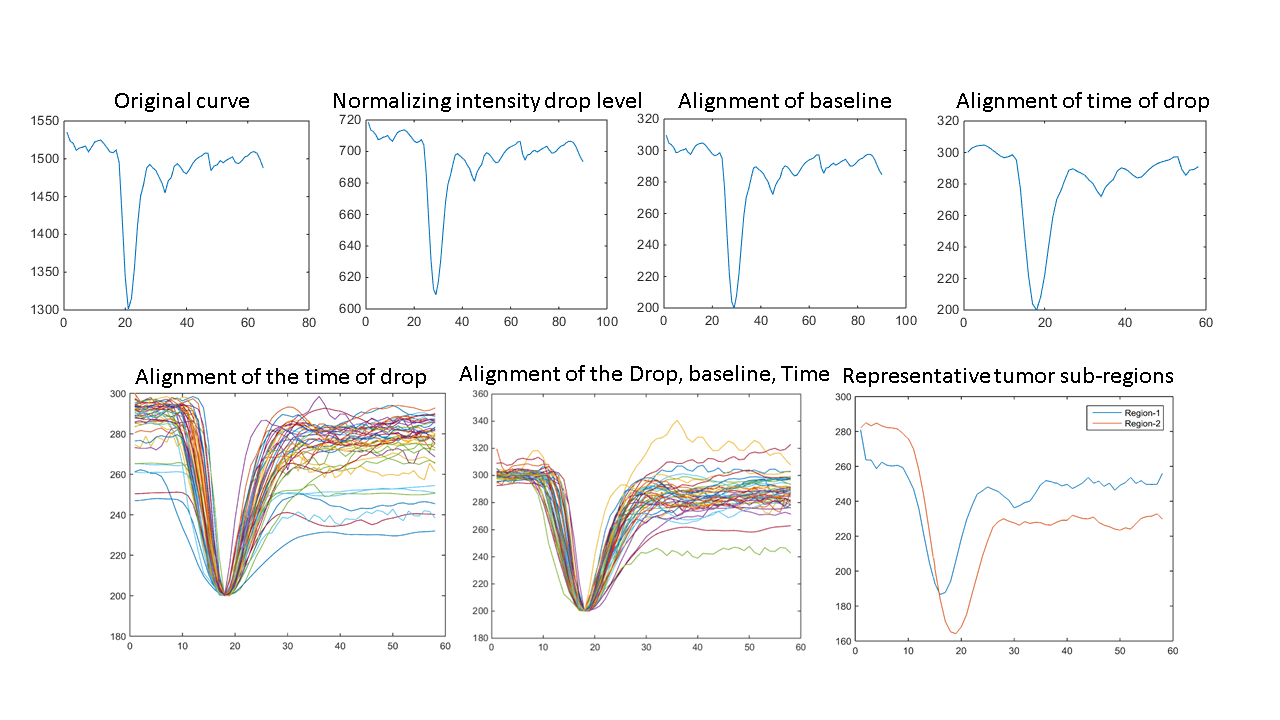


**Figure S3.** **Alignment of DSC-MRI signals as provided by neuro-CaPTk**. Top row (left-right): Original DSC-MRI curve, normalizing the drop value and making the difference of 100 between baseline and drop values, aligning the baseline value of all the DSC-MRI curves to a standard 300 value, and aligning the time of drop of the curve to standard value of 18. Bottom row (left-right): Aligned time of drop of all the DSC-MRI images, aligned baseline and intensity drop, and aligned DSC-MRI curves of two representative sub-regions of a tumor.


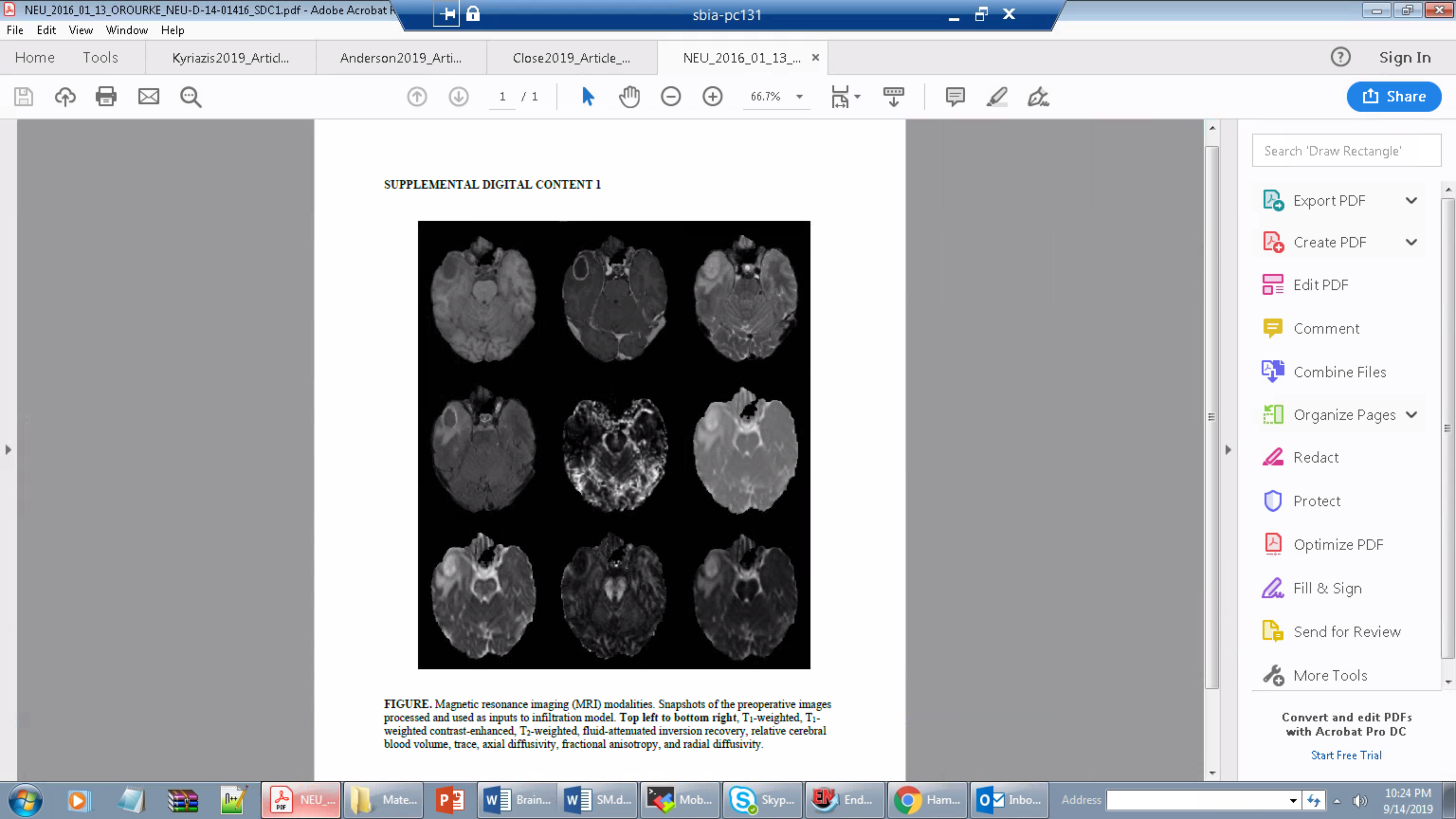


**Figure S4.** MRI modalities. Snapshots of the preoperative images processed and used to extract imaging features. Top left to bottom right, T1-weighted, T1- weighted contrast-enhanced, T2-weighted, fluid-attenuated inversion recovery, relative cerebral blood volume, trace, axial diffusivity, fractional anisotropy, and radial diffusivity.

Section S1: Specialized application modules provided by neuro-CaPTk

Current standard-of-care is generally uniform across cancer patients, however, the heterogeneous landscape and different response to the same treatment render the same-treatment-for-all approach inadequate. Embracing the concept of inter- and intra-tumor heterogeneity, an expanding part of neuro-CaPTk provides non-invasive characterization of this heterogeneity in a form personalized to each patient [^1^](#_ENREF_1). Using routines provided by neuro-CaPTk for image analysis of rich QIP signatures, earlier predictors of patient’s outcome of interest, such as survival of glioblastoma patients [^2^](#_ENREF_2), treatment response assessment in glioblastoma [^3^](#_ENREF_3), prediction of infiltration beyond the visible margins of tumors [^4^](#_ENREF_4), have been made. These modules pave a way for the enrollment of patient into clinical trials in order to increase the likelihood of detection of outcome of the treatment.

Neuro-CaPTk offers a probabilistic module [^5^](#_ENREF_5) to generate population atlases of a set of patients as a function of any key factor such as age, gender, disease characteristics, etc. These population atlases provide mapping of a new tumor to the characteristics of an existing population atlas to characterize the tumor into different disease groups. Moreover, neuro-CaPTk also allows the treating physicians to evaluate peritumoral brain tumor infiltration, thereby facilitating the physicians in intensifying of treatment areas likely to be more infiltrated while preserving critical brain structures (fiber bundles) at the same time [^4^](#_ENREF_4). To identify the critical brain structures, neuro-CaPTk provides tools to extract fiber bundles, which robustly identify the fiber bundles even in the presence of abnormal flair signal and mass effect of the tumor [^6-9^](#_ENREF_6).

Embracing the rapid growth of radiogenomics, neuro-CaPTk puts a great deal of emphasis on constructing imaging signatures of molecular markers by applying machine learning algorithms to the quantitative imaging phenomics features extracted from clinically-acquired imaging. In this context, neuro-CaPTk encompasses a similar data-driven approach to detect molecular subtypes of glioblastoma [^10^](#_ENREF_10).

Section S2: Detection of molecular markers

A. EGFRvIII mutation status

The most representative block per resected tissue specimen was chosen based on morphology and used for genetic analysis. An assay based on next-generation sequencing to detect EGFRvIII transcripts [^11^](#_ENREF_11)^,^[^12^](#_ENREF_12) was used, which was validated by Taqman reverse transcription PCR. Total nucleic acid was extracted from paraffin-embedded tissue, and complementary DNA was then synthesized from 200 ng RNA. PCR primers were designed to capture wild-type (wt-)EGFR, EGFRvIII, 3 housekeeping genes, and 3 primer sets, with increasing target sizes to assess the level of RNA degradation in the sample. The sequencing library preparation method was a 2-step PCR, with multiplex PCR followed by a second PCR to add Illumina sequencing index and adaptors. Subsequently, the sequencing library was quantified, sequenced on Illumina MiSeq, and analyzed using a bioinformatics pipeline developed in our lab. EGFRvIII presence was first evaluated by applying the following formula: EGFRvIII reads/(EGFRvIII reads + wt-EGFR reads), and then based on our results using normal brains and glioblastoma, a cutoff for EGFRvIII+ tumors was set at 5% EGFRvIII to wt-EGFR allele ratio.

**B. *IDH* mutation and 1p/19q codeletion status**

The *IDH* mutation in HUP dataset was determined by next generation sequencing and immunohistochemistry. The *IDH* mutation on HUP dataset shows *IDH1* mutation only, as *IDH2* status was not available for most of these cases. Molecular classification for TCIA cohort was derived from the 2016 pan-glioma paper [^13^](#_ENREF_13) and includes somatic mutation in *IDH (1 & 2)*, from whole-exome sequencing, and 1p/19q codeletion, from Affymetrix SNP6.0 arrays.

**References:**

**1.** Rathore S, Akbari H, Rozycki M, et al. Radiomic MRI signature reveals three distinct subtypes of glioblastoma with different clinical and molecular characteristics, offering prognostic value beyond IDH1. *Scientific reports.* 2018; 8(1):5087.

**2.** Macyszyn L, Akbari H, Pisapia JM, et al. Imaging patterns predict patient survival and molecular subtype in glioblastoma via machine learning techniques. *Neuro-Oncology.* 2016; 18(3):417-425.

**3.** Akbari H, Rathore S, Bakas S, et al. Quantitative image analysis and machine learning techniques for distinguishing true progression from pseudoprogression in patients with glioblastoma. *Cancer.* 2018.

**4.** Rathore S, Akbari H, Doshi J, et al. A radiomic signature of infiltration in peritumoral edema predicts subsequent recurrence in glioblastoma: Implications for personalized radiotherapy planning. *Journal of Medical Imaging.* 2018; 5(2):021219.

**5.** Bilello M, Akbari H, Da X, et al. Population-based MRI atlases of spatial distribution are specific to patient and tumor characteristics in glioblastoma. *NeuroImage. Clinical.* 2016; 12:34-40.

**6.** Lecoeur J, Caruyer E, Macyszyn L, Verma R. Improving White Matter Tractography by Resolving the Challenges of Edema. Paper presented at: MICCAI workshop: DTI Challenge 20132013; Nagoya, Japan.

**7.** Lecoeur J, Caruyer E, Elliott M, Brem S, Macyszyn L, Verma R. Addressing the Challenge of Edema in Fiber Tracking. *Medical Image Computing and Computer-Assisted Intervention MICCAI 2014, DTI Tractography Challenge*. Boston, MA2014.

**8.** Tunc B, Parker WA, Ingalhalikar M, Verma R. Automated tract extraction via atlas based Adaptive Clustering. *NeuroImage.* 2014; 102P2:596-607.

**9.** Tunc B, Ingalhalikar M, Parker D, et al. Individualized Map of White Matter Pathways: Connectivity-Based Paradigm for Neurosurgical Planning. *Neurosurgery.* 2016; 79(4):568-577.

**10.** Macyszyn L, Akbari H, Pisapia JM, et al. Imaging patterns predict patient survival and molecular subtype in glioblastoma via machine learning techniques. *Neuro Oncol.* 2016; 18(3):417-425.

**11.** Daber R, Sukhadia S, Morrissette JJD. Understanding the limitations of next generation sequencing informatics, an approach to clinical pipeline validation using artificial data sets. *Cancer Genetics.* 2013; 206(12):441-448.

**12.** Hiemenz MC, Kadauke S, Lieberman DB, et al. Building a Robust Tumor Profiling Program: Synergy between Next-Generation Sequencing and Targeted Single-Gene Testing. *PloS one.* 2016; 11(4):e0152851.

**13.** Ceccarelli M, Barthel Floris P, Malta Tathiane M, et al. Molecular Profiling Reveals Biologically Discrete Subsets and Pathways of Progression in Diffuse Glioma. *Cell.* 2016; 164(3):550-563.
